# Physical activity and risk of chronic kidney disease: systematic review and meta-analysis of 12 cohort studies involving 1,281,727 participants

**DOI:** 10.1007/s10654-022-00961-7

**Published:** 2023-01-10

**Authors:** Samuel Seidu, Mohammad Abdool, Abdullah Almaqhawi, Thomas J. Wilkinson, Setor K. Kunutsor, Kamlesh Khunti, Tom Yates

**Affiliations:** 1 Diabetes Research Centre, University of Leicester, Leicester, UK; 2 Hockley Farm Medical Practice, Leicester, UK; 3 Department of Family and Community Medicine, College of Medicine, King Faisal University, Dammam, Saudi Arabia; 4 National Institute for Health Research Bristol Biomedical Research Centre, University Hospitals Bristol NHS Foundation Trust, University of Bristol, Bristol, UK; 5 Musculoskeletal Research Unit, Translational Health Sciences, Bristol Medical School, Learning and Research Building (Level 1), Southmead Hospital, University of Bristol, Bristol, UK; 6 Leicester Diabetes Centre, Leicester General Hospital, Gwendolen Road, Leicester, LE5 4WP UK

**Keywords:** Physical activity, Chronic kidney disease, Cohort study, Risk factor, Systematic review, Meta-analysis

## Abstract

The role of regular physical activity in preventing vascular and non-vascular disease is well established. Chronic kidney disease (CKD) is a major cause of global morbidity and mortality and largely preventable, but it is uncertain if regular physical activity can reduce the risk of CKD. Using a systematic review and meta-analysis of published observational cohort studies in the general population, we sought to assess the association between physical activity and CKD risk. Relevant studies with at least one-year of follow-up were sought from inception until 02 May 2022 in MEDLINE, Embase, Web of Science, and manual search of relevant articles. Relative risks (RRs) with 95% confidence intervals (CIs) for the maximum versus the minimal amount of physical activity groups were pooled using random effects meta-analysis. The quality of the evidence was evaluated using the GRADE tool. A total of 12 observational cohort studies comprising 1,281,727 participants and 66,217 CKD events were eligible for the analysis. The pooled multivariable-adjusted RR (95% CI) of CKD comparing the most versus the least physically active groups was 0.91 (0.85–0.97). The association was consistent across several study level subgroups. Exclusion of any single study at a time from the meta-analysis did not change the direction or significance of the association. There was no evidence of small study effects among contributing studies. The GRADE quality of the evidence was low. In the general population, individuals who are most physically active have a lowered risk of CKD compared to those who are not or least physically active. CRD42022327640.

**Supplementary Information:**

The online version contains supplementary material available at 10.1007/s10654-022-00961-7.

## Introduction

Chronic kidney disease (CKD), conventionally characterized by the presence of kidney damage and reduced function, is a direct and major cause of global morbidity and mortality [1]. Chronic kidney disease is a major contributor to poor health outcomes of noncommunicable diseases (NCDs); it is associated with an 8–10 fold increased risk of cardiovascular disease (CVD) mortality and multiplies risk in diabetes and hypertension [2, 3]. A major societal effect of CKD is the immense healthcare costs associated with its potential outcome—end-stage renal disease (ESRD)—and the loss of productivity associated with this [2, 4]. Though risk factors for CKD vary by setting, major risk factors include diabetes, hypertension and metabolic syndrome [5, 6]. In developing countries, HIV and exposure to heavy metals and toxins play an additional role [7, 8]. Chronic kidney disease has a substantial effect on global public health and is largely preventable and treatable. Globally, in 2017, 1.2 million people died from CKD [9]. The prevalence and incidence of CKD continues to rise because of an ageing population and an increasing burden due to its major risk factors [2]. There is therefore an urgent need to identify modifiable risk factors that can reduce the risk of CKD or slow its progression.

The health benefits of physical activity are well documented and include reduction in the risk of several vascular and non-vascular diseases [10–13]. Physical activity also reduces the risk, duration or severity of infectious diseases [14, 15] and has mental health benefits [16]. Several cross-sectional studies have reported associations between physical activity and risk of CKD with inconsistent results [17–19]. Zhu and colleagues in a recent meta-analysis of 8 cross-sectional studies showed little evidence of an association between the highest vs. lowest level of physical activity and risk of CKD [20]. However, cross-sectional study designs do not address the issue of temporality. The evidence on the prospective association between physical activity and CKD is also controversial. Whereas, some studies have reported evidence of associations between physical activity and risk of CKD [21–23], others have reported no evidence of an association [24, 25]. In their recent meta-analysis, Zhu and colleagues also pooled the results of 6 observational cohort studies [20]. However, a number of relevant observational cohort studies were not included in the meta-analysis and others have since been published since this last review [20]. Furthermore, the results of these additional studies have been inconsistent. Hence, there is a need to re-evaluate the relationship in more detail. In this context, our aim was to evaluate the association between physical activity and future risk of CKD in general population settings using a systematic review and meta-analysis of all published observational cohort studies to date.

## Methods

### Data sources and searches

We registered the protocol for this systematic review and meta-analysis in the PROSPERO prospective register of systematic reviews (CRD42022327640). The conduct and reporting of this review adhered to PRISMA and MOOSE guidelines [26, 27] (Appendices 1–2). MEDLINE and Embase were searched from inception to 02 May 2022 with no language restrictions. The search strategy used a combination of MESH words or terms relating to the exposure (“physical activity”, “exercise”, “aerobic training”) and outcome (“chronic kidney disease”, “kidney failure”, “renal disease”). Details of the search strategy are presented in Appendix 3. One author (SKK) initially screened the titles and abstracts of the retrieved citations to assess their potential for inclusion. This was conducted using Rayyan (http://rayyan.qcri.org), an online bibliographic tool that helps to expedite the screening process using a process of semi-automation [28]. Full texts of the selected titles and abstracts were retrieved and detailed evaluation was done, which was independently conducted by three authors (SKK, MA and SS). To identify potential articles missed by the search of databases, manual scanning of reference lists of relevant studies and review articles was performed, and Web of Science was used to do a cited reference search.

### Study selection

We included all population-based observational cohort (retrospective or prospective designs) studies that had evaluated the relationship between physical activity and risk of incident CKD in adult general populations and reported at least one year follow-up duration for the ascertainment of outcomes. For all CKD outcomes, we accepted the range of definitions as reported by the included studies. The following studies were excluded: (i) case–control and cross-sectional studies because of their lack of temporality; (ii) those involving elite athletes and/or evaluated competitive or endurance sports; and (iii) those evaluating the associations between measures of fitness (eg, cardiorespiratory fitness, physical fitness, exercise capacity) and risk of CKD; and (iv) those conducted in people with pre-existing diseases.

### Data extraction and risk of bias assessment

Using a standardized data collection form which has been used for previous reviews of a similar nature [12, 13, 15], one author (SKK) extracted relevant data from the eligible studies and two other authors (MA and SS) independently checked the data using the original articles. We extracted data on the following study characteristics: first author surname and year of publication, geographical location, year of recruitment/baseline data collection, specific study design, demographic characteristics (age and percentage of males), sample size, duration of follow-up, physical activity type and assessment method, definition of CKD, number of CKD events, risk comparisons, the most fully-adjusted risk ratios (odds ratios (ORs), relative risks (RRs), and hazard ratios) for CKD (and corresponding 95% confidence interval [CIs]), list of covariates adjusted for, and level of adjustment (‘+’ defined as minimally adjusted analysis, i.e. age and/or sex; ‘++’ as adjustment for conventional risk factors for CKD excluding inflammation, i.e. age and/or sex plus body mass index, socioeconomic status, alcohol consumption, smoking, and comorbidities; and ‘+++” as adjustment for conventional risk factors plus inflammation). When there were multiple publications of the same cohort, we extracted data from the most comprehensive study to avoid double counting the same cohort in the pooled analysis. The criterion for selection was the one with the most extended follow-up or analysis covering the largest number of participants and events. The risk of bias within individual observational studies was assessed using the Cochrane Risk of Bias in Non-randomised Studies—of Interventions (ROBINS-I) tool [29]. The risk of bias is assessed for the following domains: confounding, participant selection, classification of interventions, deviations from intended interventions, missing data, outcome measurements, and selective reporting. For each domain, the risk is quantified as low risk, moderate risk, serious risk, or critical risk and then an overall judgement of the risk of bias is provided for each study. The Grading of Recommendations Assessment, Development and Evaluation (GRADE) tool was also used to assess the quality of the body of evidence, based on the following criteria: study limitations, inconsistency of effect, imprecision, indirectness and publication bias [30].

### Data analysis

Relative risks with 95% CIs were used as the summary measures of association. Hazard ratios and ORs were assumed to approximate the same measure of RR based on the assumptions of rare outcomes [31], consistent with previous studies [12, 13]. All studies categorised physical activity exposure (e.g., leisure-time physical activity, total or any physical activity) into user-defined categories or quantiles. Due to the varied reporting of the RR comparisons across studies, they could not be transformed to consistent comparisons (e.g., top versus bottom quantiles of the distribution of physical activity) using standard statistical methods [32–34]. However, to provide some consistency and enhance comparison and interpretation of the findings, the extreme groups (i.e., the top versus bottom or maximum versus the minimal amount of physical activity) reported for each study were used for the analyses. Several previous meta-analyses have utilised this approach [12, 13] and it is considered reliable as there is documented data that pooled estimates from transformed and untransformed data are qualitatively similar [33]. When a study reported specific types of physical activity in addition to any or total physical activity, we only used risk estimates for any or total physical activity in the pooled analysis as done for previous similar reviews [12, 13]. Relative risks were pooled using a random effects model to account for the effect of heterogeneity [35]. The extent of statistical heterogeneity across studies was quantified by standard chi-square tests and the I^2^ statistic [36, 37]. To determine the degree of heterogeneity, we also estimated 95% prediction intervals, which provide a region in which about 95% of the true effects of a new study are expected to be found [38, 39]. We explored for evidence of effect modification on the association (sources of heterogeneity) using pre-specified study-level characteristics such as geographical location, observational cohort design (prospective vs retrospective), the average age at baseline, the average duration of follow-up and number of CKD events, which was conducted using stratified analysis and random effects meta-regression [40]. To test the robustness of the observed association, we conducted a sensitivity analysis by investigating the influence of omitting each study in turn on the overall result (stata module –metaninf-). To explore for small study effects, we visually inspected constructed Begg’s funnel plots [41] and performed Egger’s regression symmetry test [42]. We employed Stata version MP 17 (Stata Corp, College Station, Texas) for all statistical analyses.

## Results

### Study identification and selection

**Fig. 1:**
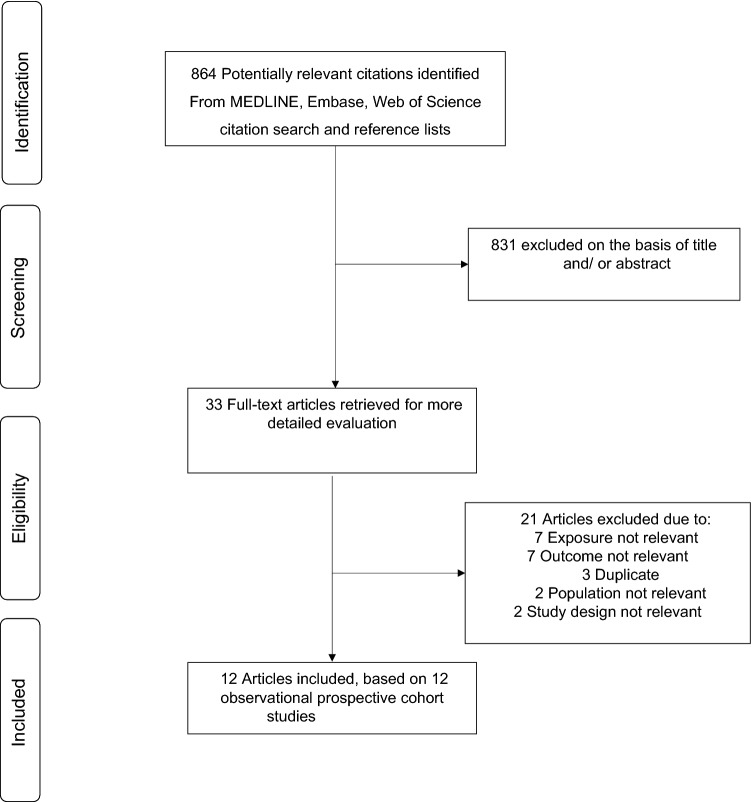
PRISMA flow diagram

We identified 864 potentially relevant citations following the search of databases, manual screening of relevant articles and Web of Science citation search. After the initial screening of titles and abstracts, 33 articles were selected for full-text evaluation. After detailed evaluation of the full-texts, 21 articles were excluded because of the following reasons: (i) exposure was not relevant (n = 7); (ii) outcome not relevant (n = 7); (iii) duplicate of a cohort already included in the review (n = 3); (iv) population not relevant (n = 2); and (v) study design not relevant (n = 2). Overall, 12 articles based on 12 unique studies comprising 1,281,727 participants and 66,217 CKD events were eligible for the review [21–25, 43–49] (Fig 1). All 12 articles were identified from the search of databases.

### Study characteristics and risk of bias

The study design and population characteristics of the eligible observational cohort studies evaluating the associations between physical activity and the risk of CKD are summarized in Table 1. The studies were published between 2003 and 2022. Ten studies were based on prospective cohort designs, with two being retrospective cohort designs [47, 48], and they were all based in general population participants. The average age of participants at baseline ranged from approximately 39 to 74 years, with a weighted mean of 47 years. All studies recruited men and women, except for one which was based on only men [47]. Six studies were based in Asia (Japan, Singapore, and Taiwan), 5 in North America (USA), and 1 in Australasia (Australia). All 12 studies assessed total physical activity exposure through self-reported or interview-administered questionnaires; the categorisation of physical activity varied across studies. The average duration of follow-up ranged from 1.0 to 24.0 years, with a weighted mean of 4.8 years. Chronic kidney disease was mostly defined as an estimated glomerular filtration rate (GFR) of <60 mL/min/1.73 m^2^ and/or proteinuria. The degree of confounder adjustment varied across studies, but all studies adjusted for established risk factors; only one study adjusted for inflammation as measured by C-reactive protein. All 12 studies were at serious risk of bias (i.e., were judged to be at serious risk of bias in at least one domain, but not at critical risk of bias in any domain) (Appendix 4).

**Table 1:** Baseline characteristics of observational studies included in review (2003–2022)

Author, year of publication	Name of study	Country	Baseline year	Males, %	Mean/median age, years	Follow-up duration, years	Physical activity comparisons	No. of CKD cases	No. of participants	Definition of CKD	Covariates adjusted for
Stengel 2003 [43]	NHANES II	USA	1976–1980	47.0	49.3	13.2	High vs low PA	189	9082	Incident CKD was defined as either treatment of end-stage kidney disease due to any cause or death related to CKD	Smoking, alcohol, BMI, age, gender, race, DM, CVD, hypertension, SBP, total cholesterol, and estimated GFR
White 2011 [44]	AusDiab	Australia	1999–2000	45.5	51.6	5.0	≥150 min/week vs 0 min/week	549	6318	Final estimated GFR of <60 mL/min/1.73 m^2^	Age, sex and kidney function at baseline
Hawkins 2015 [24]	Health ABC	USA	1997–1998	48.2	73.5	10.0	Top vs bottom third	338	2435	Incident CKD was defined as a follow-up eGFR less than 60 ml/min/1.73m^2^ 17 in individuals with baseline GFR >60 ml/min/1.73m^2^	Age, baseline GFR, sex, race, smoking status, study site, hypertension medication use, heart failure, diabetes status, pulse pressure, BMI, HDL, triglycerides, TC, CRP, and television watching
Jafar 2015 [22]	Singapore Chinese Health Study	Singapore	1993–1998	44.1	56.1	15.3	Any vs never	642	59,552	NR	Age, sex, interview year, BMI, dialect, education level, self-reported history of physician diagnosed hypertension, DM, heart disease or stroke, alcohol use, smoking, intake of ginseng, and protein intake
Foster 2015 [46]	Framingham Offspring	USA	1998–2001	45.2	59.0	6.6	Highest vs lowest PA	171	1802	Incident CKD was defined as an estimated GFR of <60 mL/min/1.73 m^2^	Lifestyle factors, age, sex, baseline estimated GFR, BMI, hypertension, DM, and dipstick proteinuria
Ogunmoroti [45] 2016	MESA	USA	2000–2002	47.2	62.0	10.2	Ideal vs poor	454	6506		Age, sex, race/ethnicity, education, and income
Michishita 2016 [47]	Fukuoka University	Japan	2008	100.0	51.6	5.0	Habitual moderate exercise vs no	23	252	Estimated GFR <60 ml/min/1.73 m^2^ and/or proteinuria	Age, BMI, smoking habit, drinking habit, estimated GFR, HbA1c levels, and systolic and diastolic blood pressure at baseline
Wakasugi 2017 [49]	SHC	Japan	2008–2009	36.9	63.7	1.0	Regular vs no regular exercise	2948	99,404	Proteinuria corresponding to ≥30 mg/dl of albumin-to-creatinine ratio	Age, hypertension, diabetes, hypercholesterolemia, smoking status, BMI, alcohol intake, regular exercise, and healthy eating habits
Guo 2020 [21]	MJ Cohort	Taiwan	1996–2014	51.6	39.1	4.2	High vs low PA	10,596	199,421	The incident CKD was identified by medical assessment. Defined as an eGFR of less than 60 mL/min/1.73 m^2^ or reported a physician diagnosis of CKD	Age, sex, education and baseline estimated GFR, physical labour at work, smoking status, alcohol consumption, vegetable and fruit intake, calendar season and calendar year, BMI, hypertension, DM, dyslipidaemia, self-report of a physician diagnosis of CVD and self-report of a physician diagnosis of cancer, and urinary protein level
Parvathaneni 2021 [23]	ARIC	USA	1987–1989	44.9	54.0	24.0	Highly active vs inactive	4820	14,537	Incident CKD defined as estimated GFR <60 mL/min/1.73 m^2^ at follow up and ≥25% decline in estimated GFR relative to baseline	Age, sex, race-center, education, smoking status, DASH diet score, diabetes, CHD, hypertension, antihypertensive medication, BMI, and baseline estimated GFR
Yamamoto 2021 [25]	J-ECOH	Japan	2006–2007	90.0	42.8	10.6	High vs inactive	4013	17,331	Incident CKD was defined as an estimated GFR of <60 mL/min/1.73 m^2^ and/or proteinuria determined using the dipstick test	Baseline estimated GFR, age, sex, smoking status, alcohol consumption, occupation, job position, overtime work, shift work, commuting mode, sleep duration, other types of physical activity, hypertension, DM, history of CVD, dyslipidemia, hyperuricemia, and BMI
Suzuki 2022 [48]	JMDC Claims Database	Japan	2005–2016	60.7	46.0	4.0	Ideal vs non-ideal	41,474	865,087	Proteinuria corresponding to ≥30 mg/g of albumin-to-creatinine ratio	Age, sex, smoking status, BMI, dietary habits, blood pressure, fasting glucose, and TC

*BMI* body mass index, *CHD* coronary heart diseae, *CKD* chronic kidney disease, *CRP* C-reactive protein, *CVD* cardiovascular disease, *DM* diabetes mellitus, *GFR* glomerular filtration rate, *HbA1c* glycated haemoglobin, *HDL* high density lipoprotein, *NR* not reported, *PA* physical activity, *SBP* systolic blood pressure *TC* total cholesterol. Study Abbreviations: *ARIC* Atherosclerosis Risk in Communities, *AusDiab* Australian Diabetes, Obesity and Lifestyle Study, *Health ABC* Health, Aging and Body Composition, *J-ECOH* Japan Epidemiology Collaboration on Occupational Health, *MESA* Multi-Ethnic Study of Atherosclerosis, *NHANES* National Health and Nutrition Examination Survey, *SHC* Specific Health Checkups and Guidance System in Japan

### Physical activity and risk of CKD

**Fig. 2:**
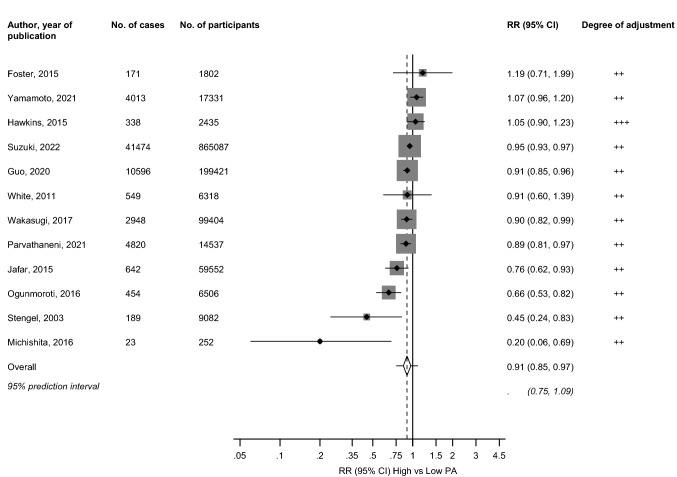
Observational cohort studies of physical activity and risk of chronic kidney disease included in meta-analysis. The summary estimate presented was calculated using random effects models and was based on fully adjusted estimates; sizes of data markers are proportional to the inverse of the variance of the relative ratio; CI, confidence interval (bars); PA, physical activity; RR, relative risk; ++, adjustment for conventional risk factors excluding inflammation, i.e. age and/or sex plus body mass, socioeconomic status, alcohol consumption, smoking, and comorbidities

In pooled analysis of 12 studies, the multivariable-adjusted RR (95% CI) of CKD comparing the most physically active versus the least physically active groups was 0.91 (0.85–0.97) (Fig. 2). The 95% prediction interval for the pooled RR was 0.75 to 1.09%, which is the range within which the true RR for any new single study will usually fall. There was substantial heterogeneity between the contributing studies (*I*^2 ^= 71%, 48 to 84%; *p *< 0.001). Exclusion of any single study at a time from the meta-analysis did not change the direction or significance of the association (Appendix 5).

### Subgroup analysis and assessment of small study effects

**Fig. 3:**
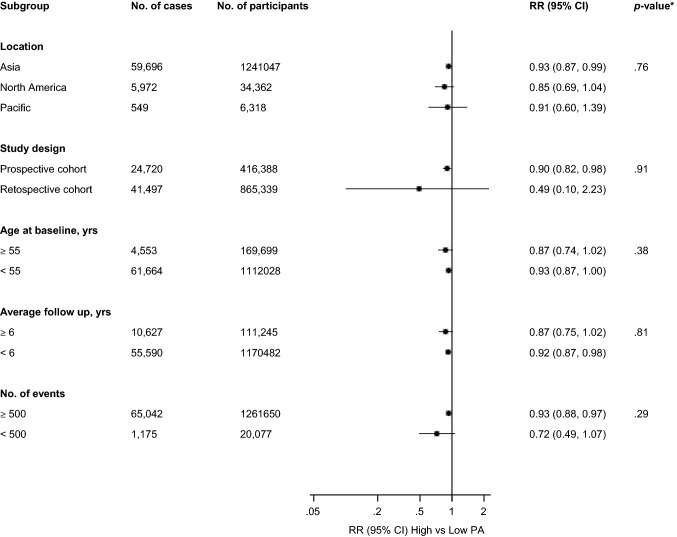
Relative risks for chronic kidney disease comparing maximal versus minimal amount of physical activity, grouped according to several study-level characteristics. CI, confidence interval (bars); PA, physical activity; RR, relative risk; *, *p*-value for meta-regression

The association between physical activity and CKD risk was consistent across several subgroups, with no significant evidence of effect modification by any of the study level characteristics (Fig. 3). A funnel plot of the 12 studies reporting on the associations between physical activity and risk of CKD showed no evidence of asymmetry (Appendix 6), which was consistent with Egger’s regression symmetry test (*p *= 0.11). Furthermore, there was no evidence of such selective reporting when studies were grouped by size in meta-regression analysis (Fig. 3).

### GRADE summary of findings

GRADE ratings for the overall incidence of CKD are reported in Appendix 7. GRADE quality of the evidence was very low.

## Discussion

### Key findings

Given the uncertainty regarding the prospective relationship between physical activity and CKD risk, we re-evaluated the association by conducting a meta-analysis of all published population-based observational cohort studies limited to general populations. In a pooled analysis of 12 observational cohort studies comprising over 1.2 million participants, comparing the most versus the least physically active groups was associated with a 9% lowered risk of CKD. The association was consistent across several relevant subgroups and in sensitivity analysis that involved recalculating the pooled estimate on exclusion of a single study at a time. The quality of the evidence was very low.

### Comparison with previous studies

The only relevant review on the topic is that by Zhu and colleagues which explored the relationship between physical activity and CKD risk using a systematic review and dose–response meta-analysis of both observational cross-sectional and cohort studies [20]. Their pooled analysis of 8 cross-sectional studies showed weak evidence of an association between physical activity and risk of CKD. Comparing the highest versus lowest level of physical activity, they observed a 16% reduced risk of CKD in pooled analysis of 6 observational cohort studies. Despite the comprehensive nature of the previous review [20], there were some limitations which included pooling estimates across cross-sectional and cohort studies in their dose–response analysis and the limited number of observational cohort studies identified despite a search end date of March 2020. The current study represents the most contemporary evidence on the relationship between physical activity and CKD risk in general population participants. Our review involved about five-fold more participants than the previous meta-analysis [20], providing more power to investigate the magnitude of the association. We showed a 9% risk reduction in CKD and our assessment of publication bias showed no significant evidence of small study bias, which was contrary to that reported by Zhu et al. [20].

### Explanations for findings

Exercise training and physical activity types such as aerobic and resistance training have the ability to positively modulate dysglycaemia, high blood pressure, obesity, dyslipidemia, and inflammation [50, 51], which are all major risk factors for CKD. Habitual physical activity may also protect against CKD via improved cardiovascular and renal endothelial dysfunction, improved insulin sensitivity, alleviation of sympathetic overactivity, slowing down the atherosclerotic process, and reduction in adipocytokines, which can damage the kidney endothelium [52–55].

### Implications of findings

The current findings on the potential for high levels of physical activity to reduce the risk of CKD add to the accumulating evidence base on the health benefits of physical activity, especially in reducing the incidence of NCDs. Current physical activity guidelines recommend a minimum of 150 min/week of moderate-intensity or 75 min/week of vigorous-intensity aerobic PA/ exercise for adults, given that these levels are associated with substantial benefits in the majority of people [56–58]. However, it is documented that many individuals do not even meet these minimum levels [59, 60]. Data on worldwide trends in insufficient physical activity from 2001 to 2016 showed that the global age-standardized prevalence of insufficient physical activity was 27.5% [61]. Given the strong link between physical activity and major NCDs, it was agreed by the World Health Organization member states that one of the ways to improve the prevention and treatment of NCDs, was to achieve a 10% relative reduction in the prevalence of insufficient physical activity by 2025 [62]. Chronic kidney disease even in its early stages is associated with extremely high morbidity and mortality, enormous economic burden and loss of productivity [2]; hence, it is a disease that warrants urgent attention. Physical activity in any form has health benefits and there is a need to promote physical activity urgently via clinical practice and population wide approaches. It has been suggested that implementing the following policies might increase population levels of physical activity in order to reduce physical inactivity by 10% by 2025: improving provision of infrastructure for non-motorised modes of transportation such as walking and cycling and encouraging their use; promoting participation in active leisure time activities; creating more opportunities for physical activity in public open spaces and parks; addressing cultural barriers that might lead to reduced participation in physical activity; and providing opportunities for safe and accessible leisure-time activities to women, who have been documented to have lower levels of physical activity [61]. Finally, though physical activity is an important strategy for the primary prevention of CKD, it may only be one piece of the puzzle. In addition to engaging in habitual physical activity, other strategies include adoption of healthy lifestyles such as consuming a healthy diet, achieving healthy weights, and avoiding tobacco use as well as beneficial modulation of modifiable risk factors such as obesity, smoking, hypertension, hyperlipidaemia and diabetes.

### Strengths and limitations

The strengths of the current evaluation include (i) the use of only observational cohort studies with at least one year follow-up, hence ensuring temporality in the association; (ii) ability to explore if the association is modified by clinically relevant study level characteristics; (iii) evidence of no significant small study effects (publication bias); (iv) assessment of the risk of bias for each individual study and the certainty of the evidence using well-established tools; and (v) sensitivity analysis to test the robustness of the association. There were several limitations, but these were mostly inherent to the studies and not the methodological approach. First, there was variation in the assessment and categorisation of physical activity exposures across studies, which could have introduced biases in our pooled results. For example, whereas some studies reported risk comparisons as high vs low, others reported it as any vs never. This did not enable transformation into consistent comparisons such as quantiles; hence comparisons could only be made between the most and least physically active. This approach is however, consistent with previous studies [12, 13, 63, 64]; it is unlikely this approach will impact the findings as there is evidence showing that pooled results from untransformed data of extreme categories are not very different from results based on transformed data [33]. Furthermore, because most studies did not quantify a unit of measurement for physical activity, a dose–response relationship could not be assessed. Second, the definition of CKD varied across studies. For instance, some studies used the estimated GFR for defining CKD, whereas others used proteinuria or the albumin-to-creatinine ratio. However, the majority of studies used estimated GFR of <60 mL/min/1.73 m^2^ and/or proteinuria. Furthermore, our leave-one-out sensitivity analysis showed our results were robust. Third, there was a potential for misclassification bias given that physical activity was self-reported. Fourth, given the varying degree of adjustment across studies, we could not evaluate the impact of a uniform approach to statistical adjustment. However, all studies adjusted for several established risk factors for CKD. Fifth, given that diabetes and CVD may exist in the causal pathway between physical activity and CKD and could be mediators, it could be argued that the pooled estimate is over-adjusted as the majority of studies adjusted for these potential mediators. However, this is unlikely given that these comorbidities are well established risk factors and potential confounders. Sixth, there was potential for small study effects [65] which is known to threaten the validity of the results in a meta-analysis [66], given that some of the smaller studies such as Stengel et al. [43] and Michishita et al. [47] reported larger effect estimates than even the larger studies. However, our assessment of publication bias (the most well-known reason for small study effects) using a variety of methods showed no evidence of small study effects. Seventh, all studies were judged to be at serious risk of bias in at least one domain of the Cochrane risk of bias tool. Finally, given the use of observational study designs with physical activity exposures assessed at baseline, there was potential for biases such as residual confounding, reverse causation, and regression dilution. None of the studies accounted for lag-time bias to minimise reverse causation. Additionally, the findings cannot be attributed to cause and effect. A meta-analysis of individual participant data with objective measures of physical activity and their repeat measures may better quantify the association between physical activity and CKD risk and ascertain if there is a dose–response relationship.

## Conclusion

In the general population, individuals who are most physically active have a lowered risk of CKD compared to those who are not or least physically active.

## Supplementary Information

Below is the link to the electronic supplementary material.

Supplementary file1 (DOCX 207 KB)

## Data Availability

All data used for the analysis are published data and publicly available.
